# No news is good news? The declining information value of broadcast news in America

**DOI:** 10.1371/journal.pone.0331607

**Published:** 2025-10-08

**Authors:** Johann D. Gaebler, Sean J. Westwood, Shanto Iyengar, Sharad Goel

**Affiliations:** 1 Department of Statistics, Harvard University, Cambridge, Massachusetts, United States of America; 2 Department of Government, Dartmouth College, Hanover, New Hampshire, United States of America; 3 Department of Political Science, Stanford University, Stanford, California, United States of America; 4 Kennedy School of Government, Harvard University, Cambridge, Massachusetts, United States of America; Toulouse Business School: TBS Education, SPAIN

## Abstract

Despite the rise of digital media, Americans are five times more likely to consume news via television than through online platforms. However, due in large part to technical hurdles, it remains unclear what content appears on broadcast news and how the mixture of content has changed over time. We consider these questions by applying a novel LLM-based approach to an understudied corpus of expert-generated summaries of virtually all news segments aired on the “big three” broadcast networks—ABC, CBS, and NBC—between 1969 and 2024. Results based on nearly one million news segments show that “information density”—the amount of time dedicated to political issues—has declined substantially over the last 50 years. Today, broadcast news spends roughly twice as much time on commercials and “soft” news and half as much on issue-based political coverage compared to a few decades ago. Since the early 1990s, the news has also shifted inward, focusing more on domestic stories and less on international affairs. These changes suggest a transformation in the informative role of broadcast news, raising questions about its impact on voter knowledge and political engagement.

## Introduction

Fifty years ago, the majority of Americans received their daily news from a single media platform: network television. The combined audience across the three networks exceeded 80 million, or roughly two-thirds of the voting-eligible electorate [1]. The dominance of network television as the primary news source began to wane with the entry of cable television in the 1980s and continued with the emergence of online news and the movement away from television to streaming. However, despite this enhanced media environment, network television news remains the single most utilized source of news for the American public, dominating online sources by a factor of five to one [2; see also 3–5].

Given the long reach of television, it is not surprising that media scholars have scrutinized the content and framing of broadcast news. Market pressures and increased competition for viewers have, unsurprisingly, led to an increase in the supply of “soft” news that focuses on celebrities, scandals, and entertainment [6,7]. In the context of political campaigns, television news tends to pay more attention to the “horse race” than the policy platforms of the competing candidates [8–10]. When networks do cover substantive issues, the framing is typically “episodic” [11], focusing on isolated events or personal stories (for instance, an individual experiencing homelessness) rather than broader societal trends (for instance, the national poverty rate). More broadly, broadcast news is frequently criticized for its lack of substantive content [12–16], which undermines its contribution to the “marketplace of ideas” and informed public discourse.

In this paper, we systematically analyze the changing content of national broadcast news since its peak during the 1960s, tracking its declining “information density”—the amount of broadcast time dedicated to important national political issues. To do so, we leverage a unique dataset containing virtually all evening news reports on the “big three” broadcast networks (ABC, CBS, and NBC) from 1969 to the present day. While previous literature has examined declines in substantive reporting over shorter spans [17], to our knowledge, this analysis represents the first such extensive longitudinal study of broadcast news content, spanning more than five decades.

Tracking the content of broadcast news over such an extended period—spanning the introduction of the draft during the Vietnam War through the 2024 presidential election—poses considerable analytical challenges. To map changes in the content of broadcast news over this period, we develop a novel LLM-based hierarchical classification scheme, which constructs meaningful categories of different granularity from the news itself in an essentially unsupervised way.

Using this classification scheme, we comprehensively analyze the long-term trends in the topics covered by the major news networks over the last half-century. We find that during this period, the content of typical news broadcasts shifts dramatically toward less substantive reports; together, human interest stories and commercials steadily account for greater and greater shares of nightly news content. In light of the continued centrality of broadcast news in American democracy, the gradual decline in the informative value of the evening news has important implications for voters’ ability to understand and participate in national political debates.

## Background and related work

### Background.

Television news has undergone significant transformations in recent decades, reflecting broader changes in technology, audience behavior, and media economics. The rise of cable news in the 1980s ushered in a 24-hour news cycle that prioritized sensationalism, immediacy, and conflict-driven narratives to maintain viewer engagement [18,19]. Concurrently, the proliferation of partisan outlets, such as Fox News and MSNBC, has deepened ideological polarization in news consumption, as audiences increasingly select sources that reinforce their political predispositions [20,21].

Technological advancements have accelerated these trends. The integration of digital platforms and social media into television news formats has shifted journalistic priorities toward real-time interaction and virality, often at the expense of depth and accuracy [22]. Moreover, the fragmentation of audiences has led to declining viewership for network news and a concentration of television news consumption among older demographics, while younger viewers gravitate toward digital-native platforms [23].

The erosion of trust in television news has further compounded these challenges. Public confidence in media institutions has declined due to perceptions of bias, sensationalism, and a failure to distinguish credible reporting from misinformation [24]. Research suggests that the rise of partisan framing and the blurring of entertainment and news formats have exacerbated these dynamics, contributing to the public’s skepticism toward traditional television journalism [25]. This shift has important consequences for democratic participation: previous work indicates that consumption of less information-dense content like entertainment television and “horse race” or strategically-framed political coverage promotes cynicism toward politics generally and reduces civic participation [26–29]; though, others argue that there is still useful information transmitted to viewers, just with less detail [30].

### Changes in broadcast news.

Several papers have looked at changes in broadcast and cable news, or other kinds of news—though typically via limited subsets of data. In a cross-platform report, Kavanagh et al. [31] examined shifts in news presentation from 1989 to 2017, focusing on broadcast television, cable news, and online platforms. The study found that cable and online outlets increasingly emphasize opinion, commentary, and emotional appeals, in stark contrast to the more neutral and fact-based presentations historically dominant in broadcast news. This evolution reflects a fragmentation of information consumption, raising concerns about how these changes affect public understanding and discourse. This work, however, spans a relatively short time frame and does not engage with additional questions about story selection, leaving open critical inquiries about the broader dynamics of agenda-setting and gatekeeping in the evolving media landscape.

Muise et al. [4] analyzed American news consumption from 2016 to 2019, revealing that approximately 17% of Americans are partisan-segregated through television, compared to about 4% online. Other work [32] has examined the impact of local television station acquisitions by conglomerate owners on news content and viewership. Analyzing data from 743 local news stations during 2017, Martin and McCrain find that ownership changes led to increased national political coverage at the expense of local news, a significant rightward shift in ideological slant, and a slight decrease in viewership.

In summary, previous research investigating changes in broadcast news has generally spanned abbreviated time periods and focused on a limited delineation of news content. Our work complements these past studies by tracking changes in broadcast news more fully and systematically over half a century.

### News classification at scale.

Techniques for categorizing the content of large news corpora have developed in an overlapping body of work in the computer science literature studying partisan bias, social media echo chambers, and related phenomena. While straightforward to scale and apply, few researchers have adopted traditional unsupervised classification methods—likely because the correspondence between categories produced by unsupervised topic modeling methods and concepts of substantive interest can be tenuous and difficult to validate [33,34]. Instead, researchers have largely relied on supervised approaches to ensure that resulting labels are relevant and conceptually coherent. In some cases, researchers have been able to leverage incidental expert classifications. For instance, Flaxman et al. [35] study ideological segregation using a corpus of over 4 million online news articles. The authors leverage the structure of news websites with identifiable news and opinion URLs to train a supervised bag-of-words model with which they identify the descriptive, “front-page” articles from the larger corpus.

Informative metadata of this kind is frequently unavailable, however, and, to date, most large-scale studies have relied on classification by human experts, bootstrapped to the larger corpus using supervised learning methods. For instance, Budak et al. [36] study media bias using a corpus of more than 800,000 online articles. To study the partisan balance of these articles, the authors first use Amazon Mechanical Turk workers to generate labels to train a supervised model identifying political news articles. To rate the partisan lean of the outlets using the approximately 100,000 identified articles, the authors recruited several hundred additional human annotators to assess the topic and ideological position of a representative subset of around 10,000 articles. Similarly, to classify the content of closed captions from news segments between 2012 and 2022 among 24 “polarizing” issues, Hosseinmardi et al. [17] use a complex two-layer human-in-the-loop classification model requiring an initial weakly-supervised classifier as well as a second stage of human annotation and supervised classification to refine the results.

The costs and difficulty associated with involving human expert labelers consequently pose substantial obstacles to understanding the large-scale evolution of news media. The new classification framework we develop here, leveraging the broad contextual knowledge and expressive capacity of LLMs, shares many strengths with classification by human experts without the associated cost and scaling difficulties.

## Data and methods

Studying the long-term evolution of broadcast news content poses both methodological and data challenges. Not only the underlying technology—including transmission and recording capabilities—but also the broader context of the news has shifted dramatically during this period, complicating the identification of coherent topics and themes applicable to five decades of social and political change. Traditional approaches like topic modeling often produce categories that, while statistically coherent, lack clear substantive interpretation or consistency across time periods. Moreover, the lack of sufficiently rich standard categories for classifying news introduces considerable researcher degrees of freedom into the creation of any idiosyncratic classification scheme, potentially undermining the validity of results. To address these challenges, we leverage an understudied corpus of news broadcast metadata covering almost the entire history of broadcast evening news, and develop a novel, unsupervised hierarchical classification framework that leverages large language models to inductively identify meaningful categories while maintaining conceptual consistency across time. (Data and code are available at https://github.com/jgaeb/no-news.)

### Data.

Our analysis is based on data from the Vanderbilt TV News Archive (VTVNA, https://tvnews.vanderbilt.edu), which has recorded all television national news broadcasts from ABC, CBS, and NBC since August 5, 1968. (We begin our formal analyses with 1969, the first full year of data.) To facilitate search and retrieval of relevant news material for researchers, VTVNA generates expert abstracts summarizing the content of each segment when they enter the archive.

In particular, each nightly news broadcast is divided into segments, with each segment corresponding to a single news story, commercial, or other “discrete” news item. Typical segments last between 10 seconds and several minutes. In addition to a brief abstract, VTVNA also annotates each segment with a variety of metadata, including the broadcaster that aired the segment and the program within which it appeared; the date and time the segment originally aired; and a brief title for the segment, indicating its contents (e.g., “U.S.-U.S.S.R. Relations/Arms Talks”).

For the majority of news segments, this abstract consists of a brief summary of the report and topics discussed written by an expert human annotator; see, e.g., S1 Fig. Beginning on August 14, 2014, segments aired by CBS and NBC, however, transitioned to an abstract consisting of the closed captions transcribing the dialogue from the segment; see, e.g., S2 Fig. (Segments aired on ABC transitioned on April 29, 2024.) Some extremely short news segments and segments for which the content is entirely contained in the title (e.g., “Introduction,” “Stock Market Report,” “Upcoming items,” “Goodnight”) do not have abstracts; neither do most commercials. Of the 1,093,885 total segments in our corpus, 626,056 have abstracts and are not commercials.

### Classification.

**Fig 1:**
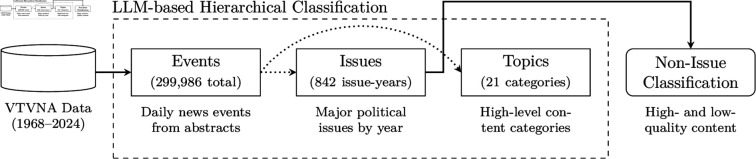
Classification pipeline. An illustration of the news classification pipeline. With LLMs, we use the VTVNA data to generate “events,” roughly corresponding to the level of granularity of a news segment. From the events we generate “issues” relevant to a year of broadcast news and “topics” covering more than five decades of news broadcasts. We further classify segments not corresponding to an issue into a variety of subtopics; see S1 Table through S4 Table for examples.

The news segments in our corpus amount to almost three years of video footage, while the abstracts summarizing these segments contain more than 43 million words. This sheer volume makes it difficult to manually review and label the entirety of the VTVNA dataset. To analyze and categorize such an extensive collection of news coverage, we use language models to develop and implement a hierarchical classification scheme, enabling systematic and scalable analysis of the corpus. The entire pipeline is shown in Fig 1.

Our classification scheme for news segments is structured into three hierarchical levels. At the most fine-grained level, we classify segments by “events”—discrete, time-bound occurrences that are the focus of individual news stories, such as a specific diplomatic meeting or an earthquake in a particular location. The number of events reported is roughly expected to match the number of news stories, acknowledging that the same event may be reported independently by different networks.

The second and more general content category associates news segments with “issues” of national importance relevant to the year the segment aired, such as healthcare reform or U.S.-U.S.S.R. relations. For ongoing issues, such as the Vietnam War, segments across multiple years can be associated with the same issue if it remained a major national concern. In any given year, we estimate that the news focuses on roughly a dozen political issues.

At the broadest level, segments are categorized into high-level “topics,” such as economics, politics, or national security. This level separates the content into broader thematic categories, allowing most news reports to be grouped under approximately two dozen overarching topics. This hierarchical threefold classification scheme enables us to systematically analyze and compare the content of broadcast news across different levels of granularity.

To develop these hierarchical classifications in a data-driven manner, we construct them using large language models (LLMs), progressing from the most granular level of events to issues and topics. (See S1 Appendix for additional prompting details and S1 Listing and S2 Listing for example prompts.)

To generate our collection of “events,” we begin by presenting a language model with the titles and abstracts from all news segments for a single day, along with instructions to generate a list of events covered that day. For each event, the model produces a one-sentence description and identifies the segments associated with it. Additionally, the model is instructed to prioritize and list the most important event of the day first.

By applying this process to each of the 19,806 “news days” in our dataset, the model identifies 299,986 distinct events. A representative sample of these events—such as diplomatic meetings, natural disasters, congressional votes, and other specific incidents—is shown in S3 Table, providing a snapshot of the granular level of detail captured by our classification scheme.

Next, we use the set of news events to generate the collection of issues. We do so sequentially, leveraging the issues generated in previous years to maintain continuity across time for events that remain salient for more than a year. In particular, we present a language model with the most important event that occurred on each “news day” in the database, together with the set of issues that have been generated in previous years. We then instruct the model to list the 10 to 15 specific issues (i.e., “inflation” rather than “the economy”) that, based on the news, viewers would think were important in a given year. (To maintain continuity across years, we also allow the model—subject to an independent correctness check—to merge issues from previous years which are substantively the same but which were given different names.) This results in 544 distinct issues—and 842 issue-year pairs—across the 50 years of data, ranging from the Vietnam War and Watergate to the 2008 Financial Crisis and COVID. A representative sample of issues is given in S2 Table.

Finally, we also leverage the collection of events to generate a list of high-level topics, again in a sequential manner. We begin with a collection of manually constructed, plausible “seed” topics. We then present a language model with a random sample of 500 events from the full dataset, pairing them with the previously generated collection of topics, or the seed topics, on the first iteration. The list of topics is then iteratively modified to better align with the sampled events, ensuring improved coverage of the dataset’s contents and achieving a balanced level of generality across all topics. Through this process, 45 candidate topic collections were generated. From the final 10 collections, the model was prompted to select the optimal set, based on criteria including abstraction, distinctness, naturalness, and comprehensive coverage. The resulting 21 topics—including Political Campaigns and Elections, International Relations and Global Policy, and Culture and Entertainment—are presented in S1 Table.

We then use a language model to classify each news segment into the issues generated for the corresponding year and the topics generated for the entire corpus. At the same time, we elicit from the model whether the segment concerns “soft” (e.g., sports, entertainment, human interest) or “hard” news (e.g., politics, economics, crime). We allow the model to leave unclassified those news segments that do not fit any of the issues or topics.

To ensure the accuracy of our model-generated labels, we compare the model-generated classifications for each of the three dimensions—viz., whether an abstract covers hard or soft news, and the topic and issue to which it most closely relates—to human experts’ categorizations. We find that model-generated labels closely match those produced by human experts, with human experts agreeing with model and with each other at roughly the same rate; see S5 Fig and S1 Appendix.

### Reclassification of non-issue-based news.

While almost all segments are assigned a corresponding topic (see S3 Fig), some news items do not correspond to political issues from the year they aired, but are nevertheless labeled “hard news” by the model. To better understand the contents of these segments, we used vector embeddings to group the relevant segments into 20 clusters using *k*-means clustering. We also manually reviewed large numbers of random samples of segments from each of the clusters, which we further split and merged, resulting in the list of sub-topics given in S4 Table, some of which overlap to varying degrees with topics. We then further reclassified each of these segments according to this schema, categorizing them as either “high-quality” (e.g., business news, government procedure, and foreign politics) or “low-quality” (e.g., the pope, animal attacks, and transportation disasters) depending on whether typical segments relating to that category of content would be expected to have substantive political importance. To ensure our reclassification is appropriately conservative, we include a number of sub-topics (including “foreign turmoil,” “crime,” “trials,” and “man-made disasters”) where some reporting is likely to be very substantive—even if the modal segment is relatively sensational—under the “high-quality” label. (Our results are relatively insensitive to the exact division between high- and low-quality subtopics; see S4 Fig and S1 Appendix for further discussion.)

## Results

**Fig 2:**
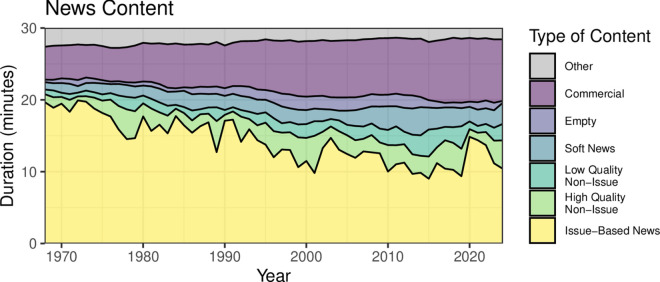
News Content. The composition of a typical 30-minute evening news broadcast between 1969 and 2024. Broadcast year is shown on the *x*-axis, and the proportion of time allotted to each type of content in minutes out of a typical 30-minute broadcast is shown on the *y*-axis. The content each band represents is indicated by the corresponding color: “other” programming not recorded in the broadcast in gray; commercials in dark purple; “empty” segments with no abstract in lavender; soft news in blue; low-quality non-issue news (see S4 Table) in turquoise; high-quality non-issue news in light green; and issue-based news in yellow. Over this 50-year period, the duration of time allotted to different types of news content changes dramatically, with increasing time spent on less substantive types of programming.

We find that the content and priorities of broadcast news have shifted dramatically over the last five decades. This trend is illustrated at the highest level in Fig 2, which depicts the allocation of airtime within a typical 30-minute newscast between 1969 and 2024. The vertical axis represents the total runtime, while colored bands delineate the proportion dedicated to distinct categories of content: (1) issue-based news addressing key national political topics of the day; (2) high-quality non-issue news, covering topics like the economy, government proceedings, foreign politics, and corruption (see S4 Table); (3) low-quality non-issue news, such as reports on natural disasters, memorials, obituaries, and anniversaries; (4) soft news, including sports, entertainment, and human-interest stories; (5) empty segments lacking abstracts, such as introductions, stock reports, farewells, and other routine elements; (6) commercials; and (7) other content, encompassing station identification, local commercials, and non-news programming omitted by VTVNA.

Over the half century depicted in Fig 2, airtime devoted to national political issues declines steadily, decreasing by approximately two minutes per decade—falling from around 20 minutes in 1969 to 10 minutes in the present day. Concurrently, the time allocated to commercials nearly doubles, increasing from roughly 4.5 minutes in the early 1970s to almost 9 minutes in the 2010s, equaling the airtime dedicated to national political issues by the end of the period.

This decline in political coverage is partially offset by growth in other content categories. Both low-quality and high-quality non-issue news expand significantly, with the former increasing from about 30 seconds to 2 minutes and the latter from 1 minute to 3.5 minutes. Similarly, airtime devoted to soft news surges from 1.5 minutes to 3.5 minutes per broadcast.

Taken together, these changes mark a profound shift in the composition of the evening news broadcasts. During the Johnson and Nixon administrations, the overwhelming majority of the 30-minute broadcast was devoted to hard news focused on national political issues. During the Biden administration, this preponderance of hard news had transformed into an almost equal division between hard news, commercials, and lower-priority or structural content.

### Changing topics of discussion.

**Fig 3:**
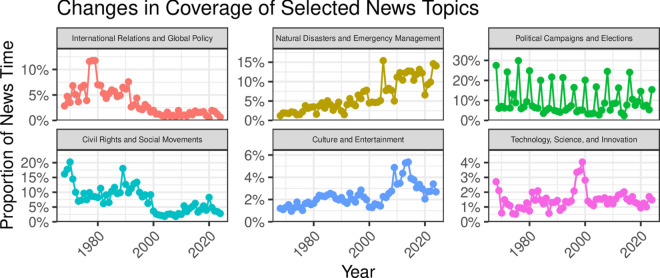
Changes in coverage of selected news topics. An illustration of the changing content of the evening news, using six of the 21 “topic” classifications. Broadcast year is shown on the *x*-axis, and the proportion of total news time (among non-commercial segments with abstracts) spent on these topics is shown on the *y*-axis. The first column highlights the declining importance of international news and news about civil rights and social movements. The second column shows the increasing salience of sensational and “soft” news topics. The third column provides evidence that topic assignments are meaningful and match expectations about changes in news content: campaign reporting increases dramatically during presidential election years, and technology reporting spikes in the late 1990s, largely covering the “electronic revolution” and concerns about the “Y2K problem.”

These dramatic changes in the evening news are also visible at the level of the topics news broadcasts cover. Each panel of Fig 3 shows the proportion of news programming devoted to six of the 21 news topics identified in our corpus: the first column shows examples of topics that have undergone declines, the second topics that have increased their share of programming, and the third topics whose time series corroborate the coherence of our classification scheme. (See S3 Fig for the full set of topics.) Our hierarchical classification scheme charts a variety of important changes in the content of the evening news over the last half-century.

Our findings show a marked decline in the coverage of international news, replaced by a growing emphasis on soft news, such as entertainment-oriented and human-interest stories. This shift reflects a significant reorientation in the priorities of news organizations. The decline in international reporting corresponds with the closure of foreign bureaus and reduced budgets for overseas coverage [15,37], making it increasingly difficult for broadcasters to cover overseas events in depth.

**Fig 4:**
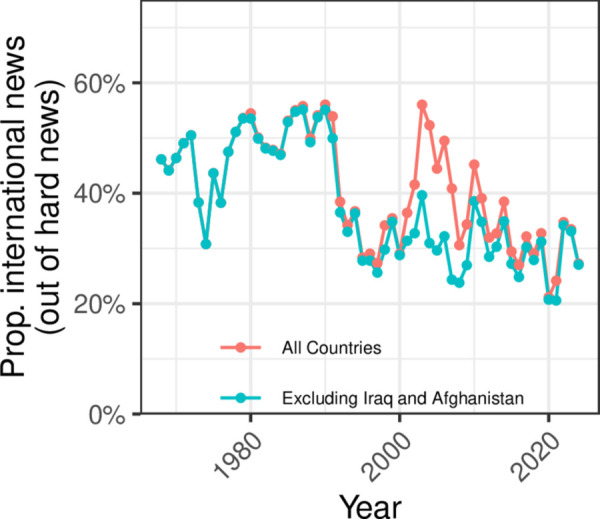
Changes in international coverage. The proportion of hard news segments mentioning a foreign country, weighted by duration. The red line indicates all foreign countries, and the blue line excludes mentions of Iraq and Afghanistan. Without these two countries, there is a durable drop of roughly 20 percentage points in the proportion of hard news mentioning a foreign country beginning in the early 1990s.

Roughly a fifth of reporting in the late 1960s and early 1970s related to the civil rights movements, as well as international social movements, such as the anti-apartheid protests in South Africa. By the mid-1970s, this declined to only about 10% of reporting, before falling precipitously again in 2000 to 5% or less of evening news content, with a brief resurgence in 2020. Similarly, reporting on international relations fell dramatically since its peak of around 10% during the Carter Administration, to virtually zero in the contemporary evening news. Since the 1990s, evening news viewers have still been exposed to discussion of foreign affairs, particularly beginning in 2001, following the September 11th attacks and the subsequent U.S. invasions of Iraq and Afghanistan. This coverage of foreign affairs, however, occurred primarily through the lens of military affairs and terrorism; see S3 Fig. The “domestication” of the evening news is visible in other ways. Between 1969 and 1991, around half of all segments mention a foreign country; that drops to just over a quarter beginning in the early 1990s, after excluding mentions of Iraq and Afghanistan; see Fig 4.

The trend toward soft and domestic news is also driven by economic pressures that compel news organizations to operate as profit centers. Soft news attracts larger audiences and generates more advertising revenue than in-depth reporting on foreign affairs [38]. As competition for viewer attention intensifies, news content has increasingly prioritized stories that align with audience preferences, often at the expense of more substantive reporting [16].

Coverage of natural disasters—shown in the top central panel—rose from a few percentage points of coverage to almost a sixth of a typical newscast in 2024. Similarly, coverage of light topics in culture and entertainment—shown in the bottom central panel—which comprised between 1% and 2% of news coverage through the mid-1990s, began to rise, roughly tripling their share of time on a typical newscast in the early 2010s.

### Shrinking coverage of political issues.

While the shift toward “softer” and more sensational news topics is significant in its own right, changes in evening news coverage of national political issues carry even greater consequences for democratic discourse. Unlike crime or human-interest stories—events that individuals might experience directly or access through local news—most voters rely on national media coverage to learn about and form opinions on critical political topics such as international trade agreements or healthcare policy [39]. The framing and prioritization of these issues in national media play a pivotal role in shaping public understanding and engagement.

As shown in Fig 3, these shifts in topical focus coincide with a stark decline in coverage of the most pressing national political issues. This reduction raises concerns about the media’s ability to inform citizens on policies and decisions central to public life and governance. The implications of this trend are far-reaching, potentially diminishing the quality of democratic deliberation and weakening the electorate’s capacity to hold leaders accountable on substantive policy matters.

**Fig 5:**
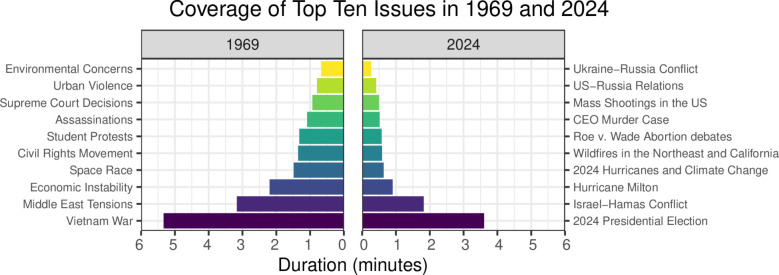
Coverage of top 10 issues in 1969 and 2024. Time devoted to coverage of the top 10 political issues during a typical newscast in 1969 vs. 2024. While the top issue in 2024 (the presidential election) received moderately less coverage than the top issue in 1969 (the Vietnam War), coverage of the remaining issues is significantly lower in 2024, ranging from roughly 15 seconds to two minutes, compared to 40 seconds to more than three minutes in 1969. In aggregate, significantly more time was spent covering top political issues in 1969 (19 minutes) than in 2024 (10.5 minutes).

The total airtime dedicated to national political issues declined sharply, from roughly 19 minutes per broadcast in 1969 to half that in 2024, the first and last full years of data in our analysis. This dramatic change is highlighted in Fig 5. The left panel illustrates the amount of time devoted to the 10 most-covered national political issues in 1969, while the right panel shows the same for 2024. In 1969, the Vietnam War dominated coverage, receiving just over five minutes of airtime during a typical evening news broadcast. By contrast, in 2024, the most-covered political issue was the 2024 presidential election, which accounted for only three-and-a-half minutes of coverage in a typical broadcast.

The disparity becomes even more pronounced for issues receiving less coverage. In 2024, the second-most-covered issue, the Israel-Hamas conflict, received just under two minutes of airtime per broadcast. This is comparable to the amount of time news broadcasts devoted to the space race in 1969, the fourth-most-covered issue. Hurricane Milton, the third-most-covered issue in 2024, received as much airtime as Supreme Court decisions, 1969’s eighth-most-covered political issue. This reduction underscores a significant narrowing of the scope of political coverage in the evening news over the past five decades.

## Discussion

By developing and applying a novel LLM-based hierarchical classification scheme, we document a dramatic decline in the substantive content of American evening news over the past five decades, with airtime devoted to national political issues falling from 20 minutes in 1969 to only 10 minutes in 2024. Concurrently, airtime for commercials has doubled, and coverage of soft news and low-quality, non-issue reporting has expanded significantly. These changes reflect a broader shift in the priorities of network news, driven by economic pressures and competition from cable and digital media. The result is a media landscape where the primary source of news for Americans is less focused on informing the public and more oriented toward entertainment.

This increasingly non-substantive content of television news is particularly concerning in the context of rising political polarization and the proliferation of misinformation. The decrease in hard news content on broadcast news may make for a citizenry that is less informed about the policies and debates that shape their lives. Historically, television news has played a critical role in fostering political engagement and enabling voters to hold leaders accountable [9,11], but the combination of market pressures and deregulation of broadcast media has led to the shift we document toward more entertainment-oriented news content.

The implications of non-substantive news programming for informed citizenship are clear. The prominence of soft news undermines classical theories of news media as key contributors to the “public sphere” [40,41]. Since television news is no longer a reliable source of public affairs information, viewers have fewer opportunities to learn about current events. Cross-national studies document a significant “knowledge gap” between Americans and residents of other industrialized democracies who demonstrate greater familiarity with current affairs at both the international and domestic levels [42–44]. Researchers attribute this discrepancy to the superior news content delivered by public broadcasters—who are not susceptible to market pressures—worldwide [44,45]. In light of hard news’s decline, Americans are at a disadvantage vis-à-vis citizens of other democracies.

We note that our work has important limitations. For instance, our airtime-based analysis cannot account for changes in how efficiently the news delivers political content, for instance through increased use of multimedia, though such gains would have to be extremely large to alter our substantive conclusions. Most importantly, our reliance on large language models (LLMs) introduces some degree of error, especially in cases where the text is difficult to evaluate due to minimal available information and ambiguity. Unlike human annotators, who often introduce errors when they are insufficiently attentive [46], LLMs are difficult to “distract,” but can hallucinate or exhibit temporal biases [47,48]. LLMs performed well overall, performing at a comparable level to human expert annotators (see S1 Appendix), but as these models evolve rapidly, our approach may need to be revised for future research. This raises questions about consistency and reproducibility, particularly for longitudinal studies. Even with these limitations, our method highlights the promise of using LLMs to analyze large-scale datasets and tackle complex social science questions in a scalable way.

The demise of substantive news programming, as documented here on the network television platform, presents a fundamental challenge to democracy at a time when it is under increasing threat from polarization and disinformation [49]. Addressing this challenge requires renewed attention to the role of network news in fostering an informed electorate. Although digital platforms provide alternative sources of information, they still lack the reach, and often the credibility, of traditional television news. The continuing substantive diminishment of broadcast news suggests that the public’s capacity to hold leaders accountable may fall, potentially weakening the resilience of democratic institutions.

## Supporting information

S1 Appendix Supplementary materials. Complete supplementary materials, including four appendix sections: Additional notes on data, Additional notes on methods, Validation study, Prompt templates. The following figures, tables, and listings are also included: **S1 Listing:** listing-system System message for the event generation prompt template; **S2 Listing:** listing-user User message for the event generation prompt template; **S1 Fig:** An example news segment abstract; **S2 Fig:** An example abstract for a segment generated using closed captions; **S3 Fig:** An illustration of the changing contents of the evening news across the 21 topic classifications; **S4 Fig:** The composition of hard news segments that do not correspond to an issue in a given year, plotted every half decade; **S5 Fig:** A comparison of the classification accuracy of the LLM classification pipeline described in the main text and human experts; **S1 Table:** Model-generated topics; **S2 Table:** A representative selection of 50 out of 842 model-generated issues in given years; **S3 Table:** A representative selection of 50 out of 299,986 model-generated events; **S4 Table:** Scheme for reclassifying non–issue-based hard news. (PDF)

## References

[1] Iyengar S. Media politics: a citizen’s guide. New York: W. W. Norton; 2022.

[2] Allen J, Howland B, Mobius M, Rothschild D, Watts DJ. Evaluating the fake news problem at the scale of the information ecosystem. Sci Adv. 2020;6(14):eaay3539. doi: 10.1126/sciadv.aay3539 32284969 PMC7124954

[3] Watts DJ, Rothschild DM, Mobius M. Measuring the news and its impact on democracy. Proc Natl Acad Sci U S A. 2021;118(15):e1912443118. doi: 10.1073/pnas.1912443118 33837145 PMC8053935

[4] Muise D, Hosseinmardi H, Howland B, Mobius M, Rothschild D, Watts DJ. Quantifying partisan news diets in Web and TV audiences. Sci Adv. 2022;8(28):eabn0083. doi: 10.1126/sciadv.abn0083 35857498 PMC9278856

[5] Prior M. The immensely inflated news audience: assessing bias in self-reported news exposure. Public Opinion Quarterly. 2009;73(1):130–43.

[6] Scott DK, Gobetz RH. Hard news/soft news content of the national broadcast networks 1972 –1987. Journalism Quarterly. 1992;69(2):406–12.

[7] Prior M. Any good news in soft news? The impact of soft news preference on political knowledge. Political Communication. 2003;20(2):149–71.

[8] Patterson TE. Of polls, mountains: US journalists and their use of election surveys. Public Opinion Quarterly. 2005;69(5):716–24.

[9] Patterson TE. Game versus substance in political news. The Oxford handbook of political communication. 2017. p. 377–90.

[10] Rothschild DM, Pickens E, Heltzer G, Wang J, Watts DJ. Warped front pages: Researchers examine the self-serving fiction of “objective” political news. Columbia Journalism Review. 2023.

[11] Iyengar S. Is anyone responsible? How television frames political issues. University of Chicago Press. 1994.

[12] Zaller J. Market competition and news quality. In: 1999 Annual Meetings of the American Political Science Association. Atlanta, GA; 1999. http://www.sscnet.ucla.edu/polisci/faculty/zaller/News

[13] Patterson TE, McClure RD. The unseeing eye: The myth of television power in national elections. New York: G. P. Putnam’s Sons; 1976.

[14] Patterson TE. Out of Order: An Incisive and Boldly Original Critique of the News Media’s Domination of America’s Political Process. Vintage; 1994.

[15] Epstein EJ. News from nowhere: television and the news. New York: Random House; 1973.

[16] Postman N. Amusing ourselves to death: Public discourse in the age of show business. Penguin; 2005.

[17] Hosseinmardi H, Wolken S, Rothschild DM, Watts DJ. The diminishing state of shared reality on US television news. arXiv preprint 2023. https://arxiv.org/abs/2310.18863

[18] Bennett WL. News: the politics of illusion. University of Chicago Press; 2016.

[19] Prior M. Post-broadcast democracy: how media choice increases inequality in political involvement and polarizes elections. Cambridge University Press; 2007.

[20] Iyengar S, Hahn KS. Red media, blue media: evidence of ideological selectivity in media use. Journal of Communication. 2009;59(1):19–39.

[21] Stroud NJ. Niche news: the politics of news choice. Oxford University Press; 2011.

[22] Boczkowski PJ. News at work: Imitation in an age of information abundance. University of Chicago Press; 2010.

[23] Pew Research Center. State of the News Media 2022 . 2022.

[24] Ladd JM. Why Americans hate the media and how it matters. Princeton University Press; 2012.

[25] Baum MA. Soft news and political knowledge: evidence of absence or absence of evidence?. Political Communication. 2003;20(2):173–90.

[26] de Vreese C. The effects of strategic news on political cynicism, issue evaluations, and policy support: a two-wave experiment. Mass Communication and Society. 2004;7(2):191–214. doi: 10.1207/s15327825mcs0702_4

[27] Cappella JN, Jamieson KH. Spiral of cynicism: the press and the public good. Oxford University Press on Demand; 1997.

[28] Hopmann DN, Shehata A, Strömbäck J. Contagious media effects: how media use and exposure to game-framed news influence media trust. Mass Communication and Society. 2015;18(6):776–98.

[29] Aalberg T, Strömbäck J, De Vreese CH. The framing of politics as strategy and game: a review of concepts, operationalizations and key findings. Journalism. 2012;13(2):162–78.

[30] Baum MA, Jamison AS. The Oprah effect: how soft news helps inattentive citizens vote consistently. The Journal of Politics. 2006;68(4):946–59.

[31] Kavanagh J, Marcellino W, Blake JS, Smith S, Davenport S, Gizaw M. News in a digital age: comparing the presentation of news information over time and across media platforms. RAND Corporation. 2019. https://www.rand.org/pubs/research_reports/RR2960.html

[32] Martin GJ, McCrain J. Local news and national politics. American Political Science Review. 2019;113(2):372–84.

[33] Ying L, Montgomery JM, Stewart BM. Topics, concepts, and measurement: a crowdsourced procedure for validating topics as measures. Political Analysis. 2022;30(4):570–89.

[34] Hoyle A, Goel P, Hian-Cheong A, Peskov D, Boyd-Graber J, Resnik P. Is automated topic model evaluation broken? The incoherence of coherence. Advances in Neural Information Processing Systems. 2021;34:2018–33.

[35] Flaxman S, Goel S, Rao JM. Filter bubbles, echo chambers, and online news consumption. Public Opinion Quarterly. 2016;80(S1):298–320.

[36] Budak C, Goel S, Rao JM. Fair and balanced? Quantifying media bias through crowdsourced content analysis. Public Opinion Quarterly. 2016;80(S1):250–71.

[37] Gans HJ. Deciding what’s news: A study of CBS evening news, NBC nightly news, Newsweek, and Time. Northwestern University Press; 2004.

[38] Baum MA. Sex, lies, and war: how soft news brings foreign policy to the inattentive public. American Political Science Review. 2002;96(1):91–109.

[39] Iyengar S, Kinder DR. News that matters: television and American opinion. University of Chicago Press; 2010.

[40] Schudson M. How to think normatively about news and democracy. Oxford: Oxford University Press; 2014.

[41] Zelizer B. From home to public forum: media events and the public sphere. Journal of Film and Video. 1991; p. 69–79.

[42] Iyengar S, Curran J, Lund AB, Salovaara-Moring I, Hahn KS, Coen S. Cross-national versus individual-level differences in political information: a media systems perspective. Journal of Elections, Public Opinion and Parties. 2010;20(3):291–309.

[43] Curran J, Iyengar S, Brink Lund A, Salovaara-Moring I. Media system, public knowledge and democracy: a comparative study. European Journal of Communication. 2009;24(1):5–26.

[44] Aalberg T, Curran J. How media inform democracy: a comparative approach. vol. 1. Routledge; 2012.

[45] Soroka S, B A, Aalberg T, Iyengar S, Curran J, Coen S. Auntie knows best? Public broadcasters and current affairs knowledge. British Journal of Political Science. 2013;43(4):719–39.

[46] Kittur A, Chi EH, Suh B. Crowdsourcing user studies with Mechanical Turk. In: Proceedings of the SIGCHI conference on human factors in computing systems; 2008. p. 453–6.

[47] Ji Z, Lee N, Frieske R, Yu T, Su D, Xu Y, et al. Survey of hallucination in natural language generation. ACM Comput Surv. 2023;55(12):1–38.

[48] Lazaridou A, Kuncoro A, Gribovskaya E, Agrawal D, Liska A, Terzi T. Mind the gap: assessing temporal generalization in neural language models. Advances in Neural Information Processing Systems. 2021;34:29348–63.

[49] Mueller RS. The Mueller report: complete report on the investigation into Russian interference in the 2016 presidential election. e-artnow. 2019.

