# Supplementary material for: No news is good news? The declining information value of broadcast news in America
Source: PLoS One. 2025 Oct 8;20(10):e0331607. doi: 10.1371/journal.pone.0331607 (PMC12507307; doi:10.1371/journal.pone.0331607)
Supplement: S1 Appendix — Complete supplementary materials, including four appendix sections: [file pone.0331607.s001.pdf]

S1 Appendix. Additional Data and Methodological Details

Additional notes on data

Our dataset was scraped from the VTVNA website with permission. In addition to the news segments from ABC, CBS, and NBC considered in our analysis, more limited data are available for MSNBC, Fox, and CNN over shorter periods; we limit our analysis to ABC, CBS, and NBC from 1969, the first complete year in our data, to the end of 2024.

In addition to segments from regular news broadcasts, many programs also consist of or include longer special reports on a single topic aired outside of the standard 6:30–7:00pm ET slot. We exclude these special segments from our analysis. The durations for segments are calculated based on manually entered timestamps which, in some cases, contain errors, leading to erroneous—and typically extremely long—durations. To avoid distorting our estimates, we exclude any segment lasting longer than 30 minutes. Together, these two exclusion criteria cover approximately 0.4% of segments in our data.

Additional notes on methods

We use a variety of LLMs as part of our data processing pipeline. We use OpenAI's `gpt-3.5-turbo-0125` fine-tuned on approximately 1,000 `gpt-4-turbo-2024-04-09` responses to our prompt, using default training parameters. To generate our collections of issues and topics, we use OpenAI's `gpt-4-turbo-2024-04-09`. Finally, to classify the segments according to this taxonomy, we use Anthropic's `claude-3-haiku-20240307`, served through Amazon's Bedrock API. In all three cases, we use the most sophisticated model available at the time of writing that can be economically employed in a particular part of the data processing pipeline. To embed the segments classified as hard, non–issue-based news, we use OpenAI's `text-embedding-large` in 256 dimensions.

Our manual reclassification of non–issue-based hard news was conducted as follows. First, vector embeddings of the relevant abstracts were clustered into 20 clusters using k-means clustering using 25 restarts. One hundred randomly chosen examples from each cluster—fifty chosen uniformly at random, fifty chosen according to distance to the cluster center—were then manually reviewed over the course of five to ten minutes per cluster to determine the cluster's contents and whether it constituted a coherent news category, or reflected other kinds of semantic similarities, as well as to assign an overall cluster label.

The twenty clusters were then redistributed among a larger number of coherent news topics. For instance, the ninth cluster ("British & European News") featured a large number of stories about the British royal family, news about the conflict in Northern Ireland, and stories about general political news in Britain and Europe more broadly; these stories roughly corresponded to the "Foreign Politics," "Foreign Turmoil," and "The Queen / British Royal Family" categories. The complete relationship between the k-means clusters and the reclassification scheme for non–issue-based hard news is as follows:

1. **Business News**: Business and Economic News (7) and Financial Markets (8);
2. **Government Procedure**: Political & Government News (2) and Domestic social and policy news (11);
3. **Foreign Politics**: British & European News (9) and International Conflict & Political News (10);
4. **Corruption**: Political and Government News (2) and Crimes & Legal News (12);
5. **Foreign Turmoil**: Military & Defense News (1), British & European News (9), and International Conflict & Political News (10);
6. **Trials**: Crime & Legal News (12), Domestic social and policy news (11), Criminal Justice & Court News (13), and Violent Crime & Public Safety News (16);
7. **Crime**: Crime & Legal News (12), Criminal Justice & Court News (13), and Violent Crime & Public Safety News (16);
8. **Man-Made Disasters**: Accidents & Disasters (3) and Environmental Disasters & Natural Hazards (20);

9. **Natural Disasters**: Natural Disasters & Severe Weather (18) and Environmental Disasters & Natural Hazards (20);
10. **Notices**: Military & Defense News (1), Death & Obituaries (5), and British & European News (9);
11. **Weather**: Weather & Meteorological News (14);
12. **Medical and Health News**: Medical & Health News (15);
13. **Transportation Disasters**: Accidents & Disasters (3), Aviation News (17), and Maritime & Ocean News (19);
14. **Animal Attacks**: Maritime & Ocean News (19);
15. **The Pope**: Catholic Church & Vatican News (4);
16. **The Queen / British Royal Family**: British & European News (9);
17. **Space Program**: Space & Aerospace News (6).

Finally, once the reclassification scheme was established, an LLM was used to classify the relevant segments accordingly.

This classification scheme is relatively insensitive to the precise classification of marginal categories: "natural disasters" are the largest single category, but including those segments under the "high-quality" label changes the overall composition by only about 10%; see S4 Figure, which shows the relative sizes of the 17 categories across the period of interest.

Validation study

To investigate the quality of our LLM-based news classification pipeline, we conducted a validation study comparing its performance to that of human experts. (Our study, protocol number IRB25-0751, was determined to be exempt from IRB review by the Harvard University-Area Committee on the Use of Human Subjects. Consent was obtained electronically, and data were analyzed anonymously.) Specifically, we randomly selected 100 segments from our corpus, which we then presented to 334 English-speaking adults located in the U.S. recruited through Prolific. We asked each participant to review the abstracts of three randomly chosen segments, determining for each segment (1) whether the segment constituted hard or soft news, (2) which topic classification best described the contents of the abstract, and (3) which issue from the year the segment aired best described the contents of the abstract. We also allowed participants to choose a "secondary" issue and topic in cases in which two possibilities were both potentially applicable. Each segment received between 7 and 11 ratings, with 63% receiving 10 ratings, and a further 21% receiving 9 ratings. We then tabulated the number of experts who chose each possibility, weighting a secondary topic or issue selection to be half of a primary selection, and designated the most commonly chosen label to be the ground truth for that segment.

S5 Figure illustrates the results of this study. Overall, our LLM-based pipeline produced results of similar quality to human experts' labels: both human experts and our pipeline chose a soft news designation that matched ground truth around 90% of the time, with the model slightly outscoring human annotators, and matched ground truth topic and issue classifications between 60% and 70% of the time. Two randomly chosen experts classified whether news was hard or soft identically slightly less than 80% of the time, and chose the same topic and issue classifications around 45% of the time. The labels produced by our pipeline matched a randomly chosen human expert's selections almost exactly as often. In line with previous news classification research [cf. 1], the genuine difficulty of placing many abstracts in a single category—and the large number of categories—seems to limit both human and machine accuracy; however, the performance of our LLM pipeline is almost precisely on par with that of human experts.

Prompt templates

The complete prompt templates for each task are available in the replication materials (https://github.com/jgaeb/no-news). The example shown in S1 Listing and S2 Listing below illustrates our prompting strategy, which involved minimal prompt engineering [2]. Our classification prompt templates generally follow the following structure: (1) a brief description of the particular classification task, (2) the structure of the response as JSON, (3) a detailed description of the fields of the structured response, (4) desired outcomes, such as the number of categories to produce, and (5) task-specific inputs, such as the collection of abstracts that appeared on a given day. In some cases, as in S1 Listing below, we also re-emphasized important desiderata, such as placing the most important event of each news day first or not repeating events after manually reviewing a small number of instances. In particular, we note that when eliciting the most important event of each news day, we rely on the classifying model's heuristic assessment of importance, rather than specifying additional criteria.

````
You summarize the day's news by giving a list of all the events that were covered by the
media, and by saying what event was the most important. You respond with a JSON object as
follows:
```json
{"events": [{"description": str, "segments": [int]}, ...]}
```
The JSON should be well-formatted, so remember to use double quotes for the keys and to
escape quotes in the strings. The string in the `description` field should be a brief
(but accurate) one-sentence summary of the event that occurred. The `segments` field
should be the ID numbers of the segments that covered the event.

In a typical news broadcast, each segment will cover a different event, so you should
expect to have anywhere from 12 to 20 events in total, unless it's an unusual day (e.g.,
slow news or one extremely important event).

It's very important that you list the most important event *FIRST*, and that you don't
list the same event more than once.
````

S1 Listing: *System message for the event generation prompt template.*

````
These are the news stories that appeared on ABC, CBS, and NBC on [DATE]:

([SEGMENT_1_ID]) [SEGMENT_1_OUTLET]
[SEGMENT_1_TITLE]:
[SEGMENT_1_ABSTRACT]
====================
...
====================
([SEGMENT_N_ID]) [SEGMENT_N_OUTLET]
[SEGMENT_N_TITLE]:
[SEGMENT_N_ABSTRACT]
====================

What *specific* events happened that day that were reported on by the media? Be sure that
your answer is a complete sentence (i.e., not just a noun or list of nouns) and specific
to the date (i.e., not 'the war in Iraq' or 'the economy', but 'Secretary Rumsfeld
announced...'). Most segments will probably report at least one event, unless it's news
analysis or human interest, but some events will be reported in multiple segments. You
should have almost as many events as segments.

Don't include the same event more than once, and list the most important event first.
````

S2 Listing: *User message for the event generation prompt template.*

US-USSR Relations / Arms Talks

(Studio) USSR noted refusing to resume arms talks with United States until Pershing 11 and Cruise missiles are removed from West Germany.

REPORTER: John Palmer

(DC) President Reagan's instructions to aides designed to obtain USSR agreement to resume arms talks outlined on screen, discussed; his perception of USSR proposal for Vienna talks on space weapons noted. Reagan's meeting last week with USSR ambassador Anatoly Dobrynin and letter to President Konstantin Chernenko recalled.

REPORTER: Emery King

S1 Fig.: *An example news segment abstract. This segment (VTVNA segment #538515) originally aired July 8, 1984 as part of the NBC Evening News.*

Tehran / Congress / James Stavridis / Trump

Now to the other major move by the president announcing in a fiery speech he is refusing to certify the Iran nuclear deal brokered during the Obama era, saying it will lead to more violence, chaos and Iran's nuclear breakout. But he is stopping short of withdrawing from the deal for now. NBC News chief foreign affairs correspondent Andrea Mitchell has more. In a blistering indictment of Iran's terrorism, starting with the hostage taking at the U.S. embassy in Tehran in 1979, the president tonigh…

S2 Fig.: *An example abstract for a segment generated using closed captions. This segment (VTVNA segment #1203952) originally aired October 13, 2017 as part of the NBC Evening News.*

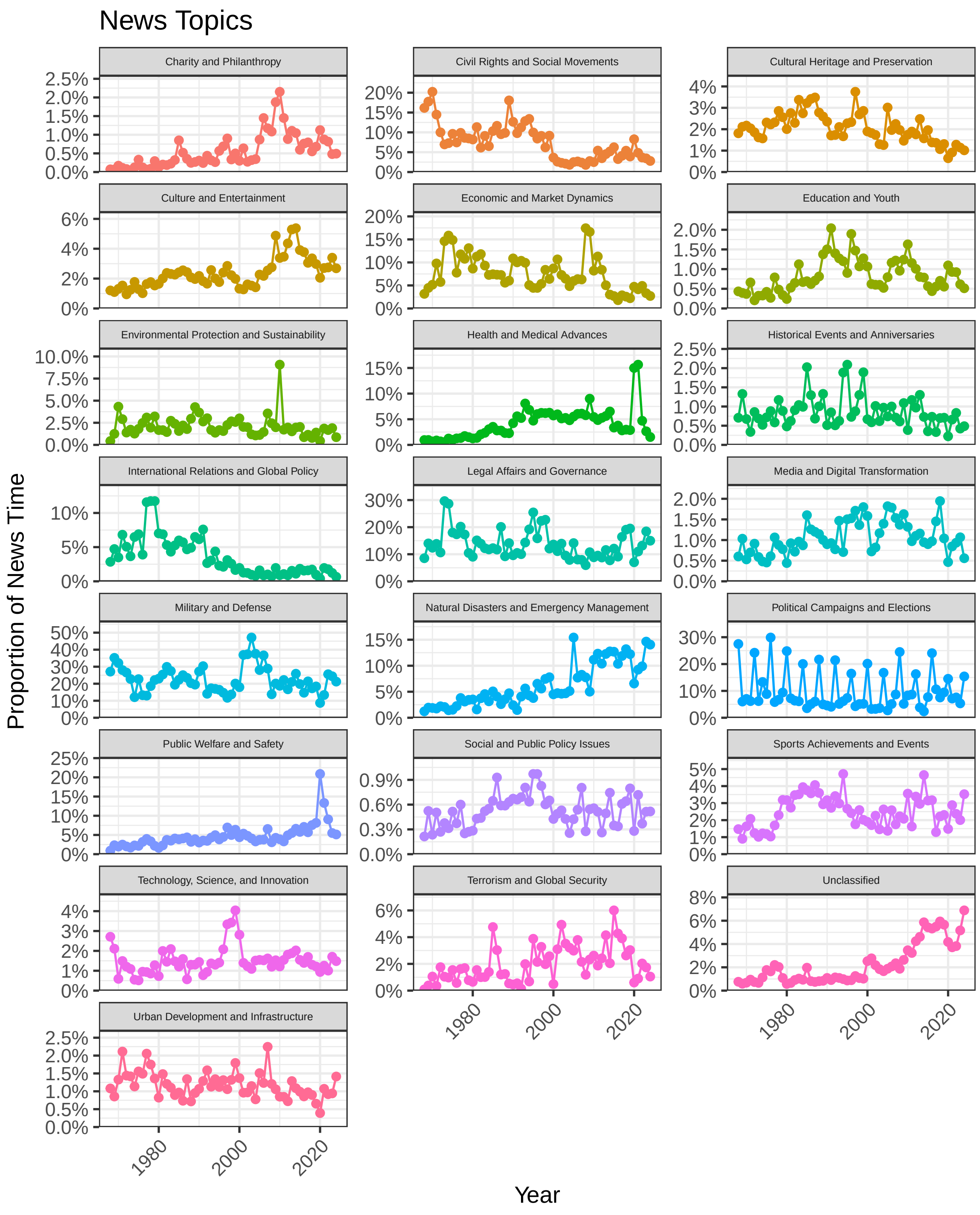

S3 Fig.: *An illustration of the changing contents of the evening news across* all *21 of the topic classifications. Broadcast year is shown on the x-axis, and the proportion of total news time (among non-commercial segments with abstracts) spent on these topics is shown on the y-axis. Segments which were not classifiable as one of the 21 topics are shown in the "unclassified" facet; the classification rate declines from roughly 99% to 95% starting in 2010.*

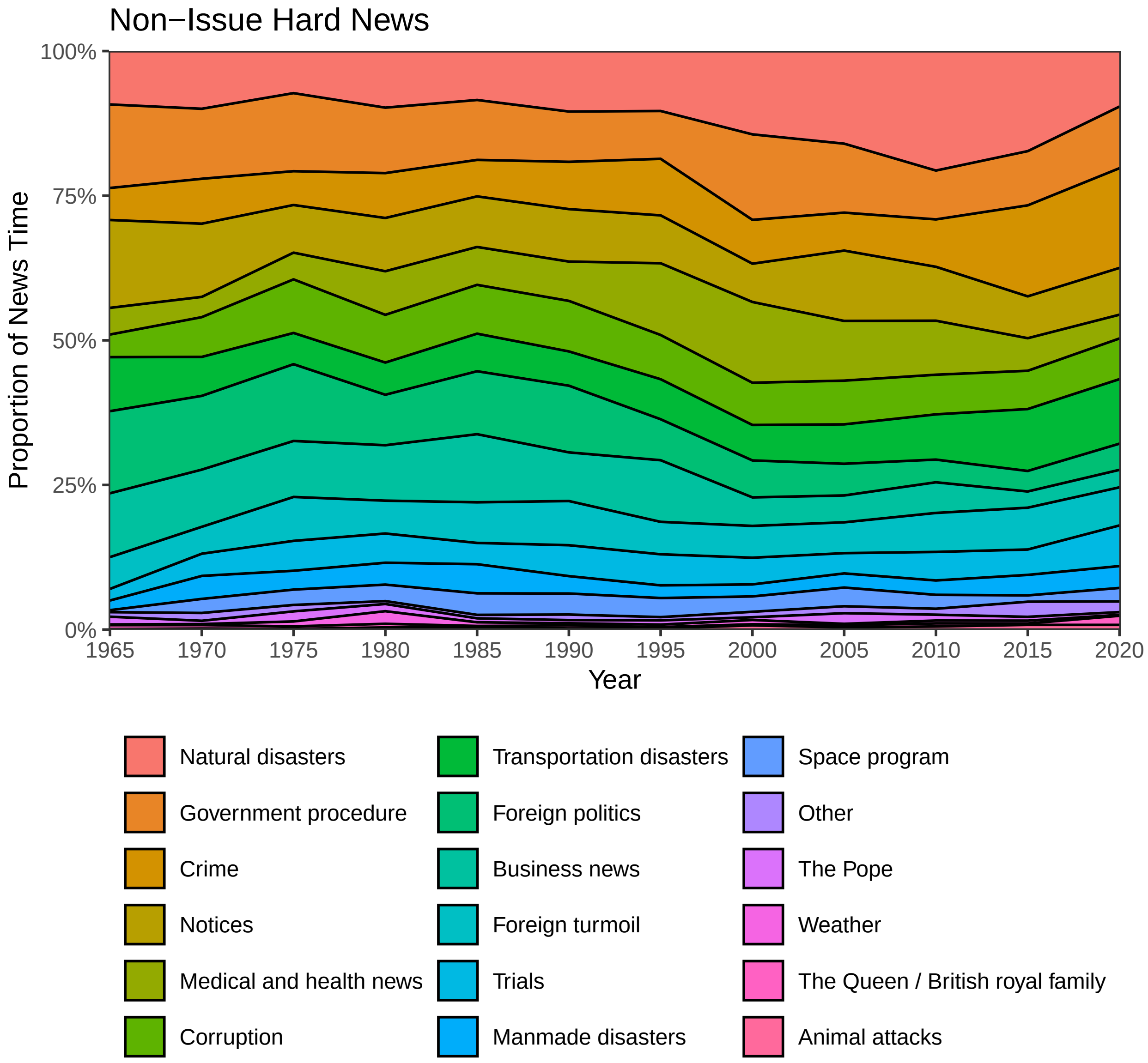

S4 Fig.: *The composition of hard news segments that do not correspond to an issue in a given year, plotted every half decade. Around 75% of segments belong to one of the eight largest categories, viz., natural disasters, government procedure, crime, notices, medical and health news, transportation disasters, foreign politics, and corruption.*

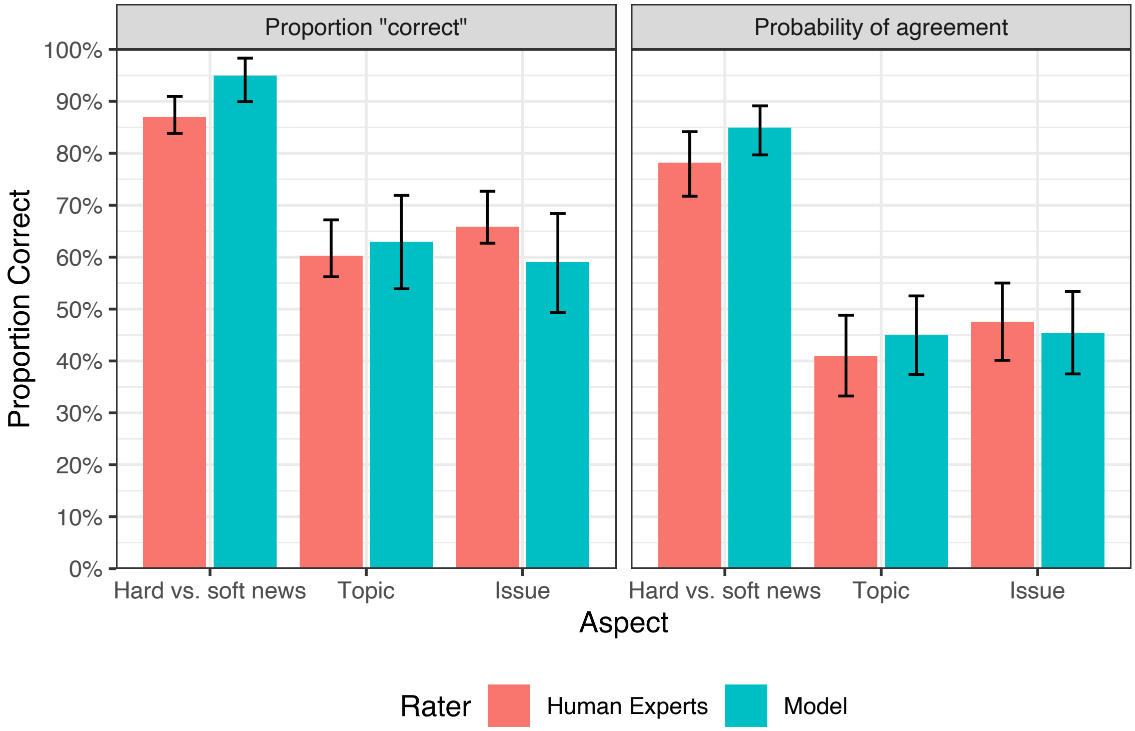

S5 Fig.: *A comparison of the performance of the LLM classification pipeline described in the main text and human experts. The left-hand panel shows the probability that the pipeline label and a randomly chosen human expert's label match the "ground truth" plurality classification for each task—viz., whether an abstract contains "hard news," and the topic and issue it most closely matches. The right-hand panel shows the probability that the labels of two randomly chosen experts (red) and the label produced by a randomly chosen expert and the LLM pipeline (blue) match. The error bars show 95% percentile confidence intervals from a two-way Bayesian bootstrap, resampling both expert raters and rated segments. Overall, the LLM pipeline and human experts perform similarly.*

S1 Table: Model-generated topics.

Topic	Description
Civil Rights and Social Movements	Covers issues and events related to civil rights campaigns, social justice movements, advocacy for marginalized groups, and significant protests aiming to change societal structures.
Military and Defense	Focuses on the activities and policies of military and defense sectors globally. This includes arms deals, military conflicts, strategic deployments, and national security concerns.
Legal Affairs and Governance	Focuses on significant legal battles, court cases, judicial outcomes, governance, policy making, and ethics disputes across various sectors. Includes high-profile trials, legal controversies, judicial rulings, governance issues, policy reactions, and ethical considerations in public and private sectors.
Media and Digital Transformation	Explores significant changes and milestones in the field of journalism and media, including the transition from print to digital, the ethical issues in journalism, the role of media in shaping public opinion, and the impact of digital transformation on news dissemination and public engagement.
Cultural Heritage and Preservation	Focuses on events and policies contributing to the preservation and celebration of cultural heritage and historical artifacts. This includes restoration efforts, debates over repatriation, and impacts of modernization on traditional cultures.
Urban Development and Infrastructure	Focuses on urban planning, development projects, infrastructure enhancements, and issues in city management, such as public transportation systems, housing, and urban policy initiatives.
Economic and Market Dynamics	Focuses on economic trends, financial markets, international trade laws, and economic policies. It includes market behaviors, financial challenges, international trade adjustments, and their impact on local and global economies.
Environmental Protection and Sustainability	Combines aspects of ecology, conservation projects, human impacts on ecosystems, pollution control, climate change, and societal responses to ongoing environmental challenges, emphasizing both local and global scales.
Charity and Philanthropy	Focuses on initiatives, both global and local, driven by philanthropic efforts, charitable organizations, and individuals aimed at improving societal conditions in various sectors like healthcare, education, disaster relief, and more.
Terrorism and Global Security	This topic covers acts of terrorism, global security threats, counter-terrorism measures, and international cooperation in security efforts. It includes news related to security breaches, terrorism-related trials, and the societal impact of terrorism.
Public Welfare and Safety	Combines issues concerning public health, consumer rights, product safety, and large-scale recalls due to safety concerns, providing a comprehensive view on matters affecting public well-being.
Culture and Entertainment	Covers news related to arts, entertainment, media, and cultural events, focusing on significant cultural festivals, achievements in cinema, music, literature, visual arts, and how media influences popular culture.
Technology, Science, and Innovation	Covers significant developments, disruptions, and breakthroughs in technology and science that transform industries, enhance consumer behaviors, and advance societal interactions. Includes discussions on digital technologies, product launches, technological infrastructures, scientific discoveries, and their implications for the future.
Education and Youth	Reports on educational reforms, school events, policies impacting youth, and stories addressing the broader societal impact on young populations.
Health and Medical Advances	Covers significant developments, policies, and controversies in the fields of medicine and health, including breakthroughs in treatment, public health crises, and debates over healthcare policies.

Natural Disasters and Emergency Management	Covers significant natural disasters like earthquakes, floods, hurricanes, and human-made disasters, including emergency response, disaster preparedness, resource management, and the socio-economic repercussions of these events.
International Relations and Global Policy	Focuses on international diplomatic relations, treaties, global policy decisions, and interactions between nations. This includes peace negotiations, international conflicts, and significant global agreements.
Social and Public Policy Issues	Examines a range of societal concerns including public health, housing, immigration, and social justice efforts, with a focus on policy reactions and public impact.
Sports Achievements and Events	Covers significant sports events, achievements, and developments within the sports industry, including major game outcomes, Olympics and international sports, shifts in sports management, and long-term impacts of sports on societies.
Political Campaigns and Elections	Covers news related to electoral processes, political campaigns, debate coverage, election results, and political endorsements. This includes both national and international election news, highlighting pivotal moments in political careers and shifts in political landscapes.
Historical Events and Anniversaries	Covers commemoration of significant historical events, anniversaries, and the ongoing impact of past events on contemporary society, reflecting on how history is interpreted and remembered.

S2 Table: A representative selection of 50 out of 842 model-generated issues in given years.

Year	Issue	Description
1971	Labor Strikes	Multiple significant strikes including those by postal workers and dock workers, impacting various sectors of the economy.
1972	International Relations and Diplomacy	The U.S. faced various diplomatic challenges and adjustments in relations with countries like China and nations involved in conflicts like the Vietnam War.
1973	Vietnam War	While U.S. direct military involvement dwindled, the focus shifted to peace negotiations, prisoner exchanges, and the aftermath implications of the conflict.
1975	Terrorism Concerns	Terrorist activities increase domestically and internationally, including bombings and high-profile kidnappings, raising security concerns.
1976	Ford's Economic Policies	President Ford faced challenges with inflation and unemployment, continuing his focus on economic stability.
1976	Steel Price Increases	Major steel companies announced price increases, causing concern over economic impacts and prompting discussions about price control.
1980	Iran-Iraq War	The conflict between Iran and Iraq escalated with significant battles over territorial disputes, affecting global oil markets and regional stability.
1982	Polish Crisis of 1981	Continued unrest in Poland following the imposition and eventual suspension of martial law, with ongoing tensions surrounding the Solidarity movement.
1983	Energy Crisis	Ongoing discussions and fears about energy sufficiency, pricing, and the transition to alternative sources continued to affect policy and market dynamics.
1983	Unemployment	While unemployment rates generally showed improvement, economic recovery remained uneven across different sectors and regions in the country.
1984	Nicaraguan Crisis	Political instability and conflicts involving the Sandinistas and the Contras highlighted Nicaragua's national crisis.
1985	Cold War Tensions and Arms Control	Ongoing negotiations and conflicts between the US and USSR including discussions on Star Wars defense system.
1986	Nicaraguan Crisis	Escalation of U.S. involvement in Nicaragua, including support for Contra rebels and rising international controversy.
1987	Supreme Court Nomination Battles	Controversy surrounded President Reagan's nominations to the Supreme Court, particularly the failed confirmation of Robert Bork and the subsequent nomination of Anthony Kennedy.
1990	Civil Rights and Legislation	Debates and legislative actions on civil rights were prominent, focusing on employment discrimination and minority protections.
1990	AIDS Epidemic	Continued efforts to combat AIDS included advancements in treatment and public health campaigns to raise awareness and reduce transmission.
1991	USA and Iraq Tensions	Continued conflict with Iraq over its noncompliance with UN resolutions and nuclear inspections, leading to continued economic sanctions and military threats.
1991	Yugoslav Wars	Violence escalated in Yugoslavia as the nation dissolved into separate entities, leading to international intervention and severe humanitarian crises.
1991	Haitian Political Instability	A military coup in Haiti ousted President Jean-Bertrand Aristide, prompting international condemnation and a refugee crisis.
1994	Haitian Political Instability	The political crisis in Haiti continued, featuring a US-led military intervention to restore President Jean-Bertrand Aristide to power.
1995	O.J. Simpson Murder Case	The O.J. Simpson trial captured national attention with its dramatic proceedings and controversial not guilty verdict, marking a pivotal moment in public discussions about race and the legal system.
1997	Princess Diana's Death	The sudden and tragic death of Princess Diana in a car crash, leading to worldwide mourning and media scrutiny.

1997	Iraq and Weapons Inspections	Continued tensions between Iraq and the United Nations over weapons inspections, leading to military buildups and diplomatic standoffs.
1997	Healthcare and Drug Approval	FDA actions on approving new drugs and treatments, including significant approvals impacting public health.
1997	Economic Instability	Global financial instability affecting major markets, including a significant impact from Asian financial crises.
1998	Clinton Impeachment	President Clinton was impeached by the House of Representatives related to his affair with Monica Lewinsky and alleged obstruction of justice.
1999	EgyptAir Flight 990 Crash	The mysterious crash of EgyptAir Flight 990 into the Atlantic Ocean led to extensive investigations suspecting possible deliberate actions by the co-pilot.
2000	Energy Crisis and California Blackouts	Utility deregulation led to a significant energy crisis in California, characterized by rolling blackouts and one of the state's largest utility companies facing bankruptcy.
2001	September 11 Attacks	Terrorist attacks involving the hijacking of four planes resulted in devastating crashes into the World Trade Center, the Pentagon, and in Pennsylvania, leading to significant loss of life and a global reevaluation of security measures.
2002	Homeland Security Department Creation	Following the September 11 attacks, a new Department of Homeland Security was created to consolidate various agencies and bolster domestic security.
2003	Environment and Climate Policy	Environmental issues were highlighted by policy changes, including the government's handling of forest management and discussions on emissions standards.
2007	Product Recalls	Significant recalls of children's toys, pet food, and other products sparked concerns about safety standards, particularly for goods manufactured in China.
2008	2008 Presidential Election	The 2008 presidential election marked a historic victory as Barack Obama became the first African American president of the United States.
2010	Health Care Reform	The implementation and legal battles over the health care reform law, signed by President Obama, continued to influence national debates.
2011	Japanese Earthquake and Fukushima Nuclear Disaster	A massive earthquake and tsunami struck Japan, leading to severe nuclear accidents at the Fukushima Daiichi Nuclear Power Plant.
2012	Eurozone Crisis	Ongoing financial instability in European countries such as Greece and Spain, requiring extensive bailouts and economic reforms.
2013	Climate Change Initiatives	Increased national and international efforts to address climate change, including debates over policy approaches and the impact of new environmental regulations
2014	US-Cuba Relations	The US and Cuba agreed to normalize diplomatic relations after more than fifty years, marking a significant shift in international relations.
2016	Syrian Civil War	The Syrian Civil War escalated with continued international involvement and significant humanitarian crises, including the battle for Aleppo.
2016	Cybersecurity and Data Breaches	Significant concerns about cybersecurity were highlighted by multiple data breaches, including a substantial attack on Yahoo accounts.
2017	Las Vegas Mass Shooting	A gunman killed 58 people in Las Vegas in the deadliest mass shooting in modern US history.
2018	Government Shutdown	A partial government shutdown was triggered by disagreements between President Trump and Congress over funding for a border wall.
2019	Climate Change Actions	Global climate strikes and discussions highlighted urgent calls for action to address climate change.
2020	Wildfires in the West	Massive wildfires, mostly in California, Oregon, and Washington, resulted in significant destruction and raised concerns over climate change and forest management practices.

2021	Racial Justice and Policing	Continued national focus and debates on policing, racial injustice, and the judicial outcomes of high-profile case trials like Derek Chauvin and Ahmaud Arbery.
2021	Vaccine Mandates and Public Health Policies	Widespread discussions and policies about vaccine mandates and public health safety measures to combat COVID-19, leading to political and social disagreements.
2021	2021 Capitol Riot	The January 6th attack on the U.S. Capitol by supporters of President Trump attempting to overturn the election results led to deaths, injuries, and ongoing national security concerns.
2022	Global Energy Crisis	Rising energy costs and supply chain issues related to geopolitical tensions and post-pandemic recovery affected global economies and policies.
2022	Climate Change and Environmental Disasters	Severe weather events and natural disasters, including hurricanes and unprecedented winter storms, underscored ongoing concerns about climate change.
2023	Energy Policy Debates	Global discussions on energy sustainability, oil dependency, and transitions to renewable resources intensified amid climate concerns.

S3 Table: A representative selection of 50 out of 299,986 model-generated events.

Date	Description
1972-06-22	Militant wing of the IRA offers a cease fire in Northern Ireland, with conditions that will start on Monday.
1973-01-04	North Vietnam states that United States bombing raids of Hanoi area killed 1300 people and wounded 1260.
1973-07-25	The House Appropriations Committee killed the Special Projects Fund amid speculation over its potential connection to funding Watergate burglars.
1974-12-16	The Senate passes a bill making Little League available to all young people, not just boys.
1975-04-20	Cattle in Michigan are still dying a year after a poison chemical, PBB, was mistakenly mixed with their feed, with no antidote for PBB and concerns about its effects on human consumption.
1976-02-24	Japanese police raid homes and businesses of those allegedly involved in the Lockheed scandal, including the residence of Lockheed agent Yoshio Kodama.
1977-04-22	China's Vice-Premier Li Hsien-nien is quoted as saying that World War III is inevitable due to the US-USSR arms race, declaring the USSR as China's most serious enemy.
1977-12-21	President Sadat and Israeli defense minister Ezer Weizman met to discuss a comprehensive Middle East settlement and the upcoming meeting between Sadat and Begin, with talks focusing on Israeli proposals for the West Bank and Sinai territories.
1979-03-20	An Exxon refinery fire in Linden, New Jersey, was reported.
1981-09-25	Sandra Day O'Connor was sworn in as the first female Supreme Court Justice.
1982-12-19	The effects of steel industry cutbacks on miners in the Iron Range of Minnesota were highlighted.
1983-05-02	Severe storms in the Midwest resulted in a significant death toll, with tornadoes causing widespread damage in several states including Ohio, Indiana, and Michigan.
1983-05-03	Catholic bishops' conference supported the nuclear freeze movement in a pastoral letter to be voted on soon.
1986-07-11	The growing popularity of gourmet ice cream, including its economic impact, is explored.
1987-02-10	President Reagan's support for a bill banning federal aid to organizations supporting abortion was acknowledged by White House aides.
1987-10-30	The life of farmer's son Osband Sargeant in New London, New Hampshire, was featured, focusing on his purchase of the town's common green.
1988-03-24	A peace agreement between Sandinistas and Contras in Nicaragua was recalled, and Hondurans' response to the United States military presence was featured.
1988-10-15	A senior CIA official stated that Mikhail Gorbachev has little chance to rejuvenate the Soviet economy and could be in political trouble in the USSR.
1990-09-28	The index of leading economic indicators showed a drop for the last month, indicating a weakening economy.
1990-10-08	President Bush and Congressional leaders continue negotiations on a budget agreement, with the Senate working on passing the House's budget package.
1993-02-12	Jack in the Box restaurants decided to change public relations firms following the tainted meat scandal.
1994-12-15	Senator Phil Gramm and other Republicans in Congress reacted to President Clinton's tax plans.
1995-06-30	Supplies were transferred from the space shuttle Atlantis to the Russian space station Mir.
1995-09-30	The Victim Information and Notification Everyday (VINE) automated program in Jefferson County, Kentucky, to notify crime victims of criminals' moves through the justice system, was featured.
1997-08-04	England celebrated the 97th birthday of the Queen Mother.
2000-02-29	A mudslide in Sao Paulo, Brazil, followed torrential rains.
2003-02-12	Astronauts on the International Space Station commented on the breakup of the space shuttle Columbia and the ongoing situation on Earth.

2003-11-14	Republicans in the House and Senate reached an agreement on a major overhaul of US energy policy.
2003-12-12	Britain's knighting of rocker Mick Jagger reported.
2004-12-21	The popularity of televised poker games among kids raised concerns about potential gambling addiction.
2007-11-09	NASA faced criticism for throwing a massive awards party while complaining of being under-funded.
2008-12-09	Federal Reserve Board chairman Ben Bernanke stated opposition to using central bank money for the auto industry bailout.
2009-06-15	The 5th grade choir from P.S. 22 in New York City featured for their performances, with commentary on how the program impacts students.
2010-01-26	A report on the issue of cruelty to animals at milk factory farms, including the practice of tail docking of cows, is examined.
2011-12-16	A report from Japan's government stated that the Fukushima nuclear plant is finally stable.
2012-01-01	Britain's Prince Philip made his first public appearance after being hospitalized for a heart procedure.
2013-01-25	The spread of a new strain of contagious norovirus across the country is reported, with stomach flu statistics cited and details given about how to deal with it.
2013-04-23	Elvis impersonator Paul Curtis was released in the ricin mail case after further investigation.
2013-05-15	The town of Green Bank, West Virginia, enforced 'radio silence' to allow the Green Bank Radio Telescope to listen to sounds in space.
2013-12-19	Concerns were raised about the safety of chicken in the United States, with calls for more government testing.
2014-06-08	An aerial parachute tribute in France marked the 70th anniversary of D-Day in World War II.
2015-12-08	Fired USC college football coach Steve Sarkisian filed an alcohol disability lawsuit against the university.
2017-03-13	Ray Chavez, the oldest surviving veteran of Pearl Harbor, celebrates his 105th birthday with no sign of slowing down.
2018-01-29	A winter storm threatens parts of the U.S., including two separate systems being tracked.
2019-05-12	The White House opposes Congressional Democrats' subpoenas and investigations, including those related to Trump's tax returns and the Mueller report.
2022-12-01	Health concerns are growing in Hawaii as the eruption at Mauna Loa threatens to cut off the main highway, spewing ash and acidic gases into the air.
2023-07-19	A U.S. soldier, Travis King, defected to North Korea by crossing the border from the DMZ and was detained.
2023-08-06	A boat crash off Italy's Amalfi Coast killed American publishing executive Adrienne Vaughan.
2023-09-10	Spanish soccer official Luis Rubiales resigned after a public kiss with player Jenni Hermoso following Spain's World Cup victory caused controversy.
2024-10-26	Former President Donald Trump campaigned across two battleground states, highlighting key issues and seeking support ahead of the election.

S4 Table: *Scheme for reclassifying non–issue-based hard news.*

Subtopic	Description
**High-Quality Non-Issue News**	
Business news	Stock market reports, mergers and acquisitions, SEC investigations, stock buybacks, strikes and labor issues, etc.
Government procedure	Presidential, congressional, and judicial appointments, recesses, vacations, resumptions, etc.
Foreign politics	Foreign elections, diplomatic events, and other "peaceful" political news from foreign countries.
Medical & Health News	New drugs and medical technology, medical and public health research, disease outbreaks, etc.
Corruption	Reports of government and private corruption, financial scams and racketeering, and bribery.
Foreign turmoil	Riots, terror attacks, crises, and disorder in foreign countries.
Trials	High profile criminal and occasionally civil trials.
Crime	Reports of murders and other violent crimes, shootouts, prison breaks, kidnappings, whereabouts of serial killers, etc.
Man-Made Disasters	Oil spills, toxic dumping, industrial accidents, fires.
**Low-Quality Non-Issue News**	
Natural Disasters	Extreme weather (hurricanes, floods, tornadoes, typhoons, blizzards, etc.), earthquakes, volcanic eruptions, wildfires, landslides and avalanches, etc.
Notices	Memorials, anniversaries, dedications, especially military; deaths, health status, and retirements of elder statesmen, celebrities, etc.
Weather	Conventional weather reports for different parts of the U.S.
Transportation Disasters	Plane crashes, train derailments, barges crashing, ferries sinking, etc.
Animal attacks	Shark attacks, stingray attacks, bear attacks, etc.
The Pope	Papal visits, encyclicals, etc.
The Queen / British Royal Family	Royal weddings, births, deaths, scandals, etc.
Space Program	Shuttle launches, new space technology, reports on probes, landers, etc.

References

[1] Budak C, Goel S, Rao JM. Fair and balanced? Quantifying media bias through crowdsourced content analysis. Public Opinion Quarterly. 2016;80(S1):250–271.

[2] Liu P, Yuan W, Fu J, Jiang Z, Hayashi H, Neubig G. Pre-train, prompt, and predict: A systematic survey of prompting methods in natural language processing. ACM computing surveys. 2023;55(9):1–35.
